# Effects of sex, age, and visits on receipt of preventive healthcare services: a secondary analysis of national data

**DOI:** 10.1186/1472-6963-6-15

**Published:** 2006-02-23

**Authors:** Anthony J Viera, Joshua M Thorpe, Joanne M Garrett

**Affiliations:** 1 Robert Wood Johnson Clinical Scholars Program, University of North Carolina at Chapel Hill, Chapel Hill, NC, USA; 2 School of Nursing, Duke University, Durham, NC, USA; 3 Robert Wood Johnson Clinical Scholars Program and Department of Obstetrics/Gynecology, University of North Carolina at Chapel Hill, Chapel Hill, NC, USA

## Abstract

**Background:**

Sex and age may exert a combined influence on receipt of preventive services with differences due to number of ambulatory care visits.

**Methods:**

We used nationally representative data to determine weighted percentages and adjusted odds ratios of men and women stratified by age group who received selected preventive services. The presence of interaction between sex and age group was tested using adjusted models and retested after adding number of visits.

**Results:**

Men were less likely than women to have received blood pressure screening (aOR 0.44;0.40–0.50), cholesterol screening (aOR 0.72;0.65–0.79), tobacco cessation counseling (aOR 0.66;0.55–0.78), and checkups (aOR 0.53;0.49–0.57). In younger age groups, men were particularly less likely than women to have received these services. In adjusted models, this observed interaction between sex and age group persisted only for blood pressure measurement (p = .016) and routine checkups (p < .001). When adjusting for number of visits, the interaction of age on receipt of blood pressure checks was mitigated but men were still overall less likely to receive the service.

**Conclusion:**

Men are significantly less likely than women to receive certain preventive services, and younger men even more so. Some of this discrepancy is secondary to a difference in number of ambulatory care visits.

## Background

The two overarching goals of Healthy People 2010 are to increase quality and healthy years of life and eliminate health disparities. Recognizing that the health disparity between men and women is not simply biological, the agenda of Healthy People 2010 calls for greater attention to and scientific exploration of the health differences between men and women [1].

Several studies have identified factors associated with disparities in the use and/or receipt of preventive services [2-20]. Many of these studies have examined the influence of enabling resources (e.g., insurance, usual source of care) on preventive services use [4,8,10,11]. Others have focused on differences in receipt of preventive services by characteristics such as race/ethnicity or by community (rural vs urban) [5,6,4,15,17,18,20]. Few studies have addressed the disparity in receipt of preventive services between men and women.

Like sex, age influences the receipt of preventive services, with younger individuals less likely to receive services [21]. In the United States in 2002, women 25 to 44 years of age accounted for 17 percent of all office-based visits to healthcare providers [22]. Visits for routine Pap smears and other reproductive needs, including pregnancy, provide multiple opportunities for health professionals to incorporate other preventive services as deemed appropriate. Younger to middle-aged men lack similar impetus to visit healthcare providers. We therefore hypothesized that sex and age would exert a combined effect on receipt of preventive services and that this combined effect would be accounted for by the difference between men and women in their number of ambulatory visits.

## Methods

### Medical Expenditure Panel Survey (MEPS)

MEPS is a longitudinal survey that provides nationally representative estimates of healthcare use and expenditures for the U.S. civilian non-institutionalized population. The 2000 MEPS consisted of 12,280 households, comprised of 23,839 individuals, from the 1998–1999 National Health Interview Survey. For purposes of this analysis, year 2000 data were collapsed for a cross-sectional sample of the population. After excluding individuals less than 20 years of age, over 14,900 adults were included in these analyses.

### Variables

Patient sex served as the main independent variable. Men and women were stratified into five age groups. For adjustment purposes, other independent variables were selected based on the Andersen model and prior studies demonstrating influence on receipt of preventive services [5,6,8,9,14,17,23]. Selected covariates were: race/ethnicity, education, marital status, income, insurance, usual source of care, perceived health status, and region of country. Number of ambulatory care visits was operationalized as whether or not individuals had one or more outpatient or office-based visits in the previous year.

From the MEPS data file, we selected items representing five preventive services applicable to both men and women: whether a doctor has checked respondent's blood pressure within last two years, duration since respondent's last cholesterol measurement, whether a doctor has advised respondent (if a smoker) to quit smoking within the past 12 months, duration since last blood stool test home kit, duration since last sigmoidoscopy or colonoscopy, and duration since last routine checkup. Answers to these items were by self-report.

For our blood pressure screening variable, we excluded respondents who reported that they had been previously diagnosed with hypertension. Those remaining who reported that they had a blood pressure measurement within the past two years were considered to have received this preventive service. For our cholesterol screening variable, we excluded respondents already diagnosed with hyperlipidemia and considered those who reported a cholesterol measurement within the last five years to have received this preventive service. To create a colorectal cancer screening variable, we considered those over age 50 who reported a blood stool test home kit within the past year, or a sigmoidoscopy or colonoscopy within the past five years to have received appropriate screening [24]. We considered those who reported a checkup within the past two years to have received this service. These intervals are also commonly used in other studies of receipt of preventive services [6,11,14,17].

### Statistical Analysis

The statistical software STATA 8.0 (STATA Corporation, College Station, TX) was used for all analyses with incorporation of appropriate sampling weights, primary sampling units, and strata to account for the complex survey design. Weighted percentages of men and women within each age group as well as within categories of selected covariates were determined. For bivariate analyses, weighted percentages were determined by 2 × 2 tables and tested for significance using chi-square comparing sex to each outcome, and then were stratified by age category. Unadjusted odds ratios and 95% confidence intervals were determined from logistic regression models without covariates.

Multivariable analyses were conducted using separate logistic regression models for each outcome, with sex as the main independent variable adjusted for covariates. The five age categories were modeled using four dummy variables with the oldest age category left out as the reference category. To evaluate for interaction between sex and age (i.e., stratification by age), interaction terms between sex and each of the four age category dummy variables were added to the models. A four degree-of-freedom Wald test was used to test for the interaction effect. A significant test means that the odds ratios comparing men to women differed by age group. An exponentiation of the linear combination of the beta estimates was used to estimate adjusted odds ratios for the main exposure (sex) and each of the four interaction terms (sex by age dummy variables). The variance-covariance matrix was used to estimate 95% confidence intervals around each odds ratio [25,26]. Finally, to test whether interaction between sex and age on receipt of preventive services was accounted for by group differences in outpatient care-seeking, the Wald test was re-run after the ambulatory care visit variable was added to the models.

## Results

In the year 2000, approximately 48 percent of the nation's adult population was men, almost two-thirds of whom were between the ages of 20 and 49 (Table 1). Men were more likely to be uninsured and lack a usual source of care. Men were also slightly more likely to be college graduates and report their health as excellent.

Table 2 shows unadjusted and adjusted odds ratios (and 95% confidence intervals) for the receipt of the selected preventive services by men compared to women. Men were significantly less likely than women to receive each of the selected services with the exception of colorectal cancer screening. Table 2 also shows that the adjusted odds ratios differed little compared to the unadjusted odds ratios, suggesting minimal confounding due to the covariates.

**Figure 1:**
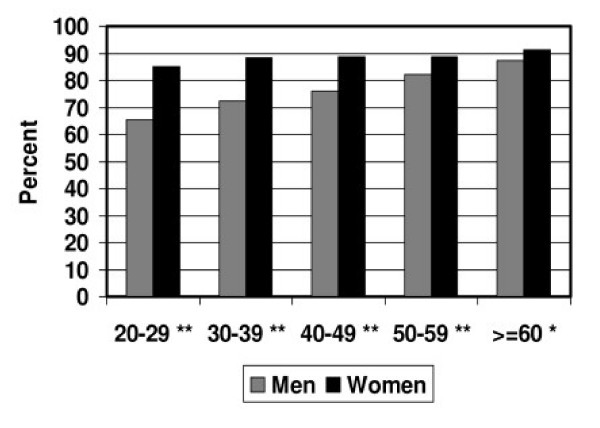
**Blood pressure check within two years (excluding known hypertensive individuals)**. Graph shows unadjusted weighted percentages of receipt of preventive services by age groups. ** p < .01, * p < .05.

**Figure 2:**
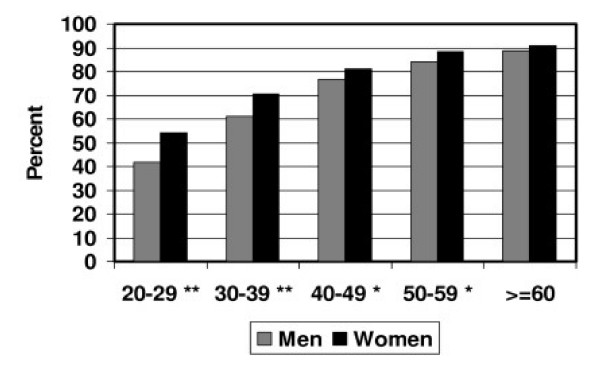
**Cholesterol check within five years (excluding known hyperlipidemic individuals)**. Graph shows unadjusted weighted percentages of receipt of preventive services by age groups. ** p < .01, * p < .05.

**Figure 3:**
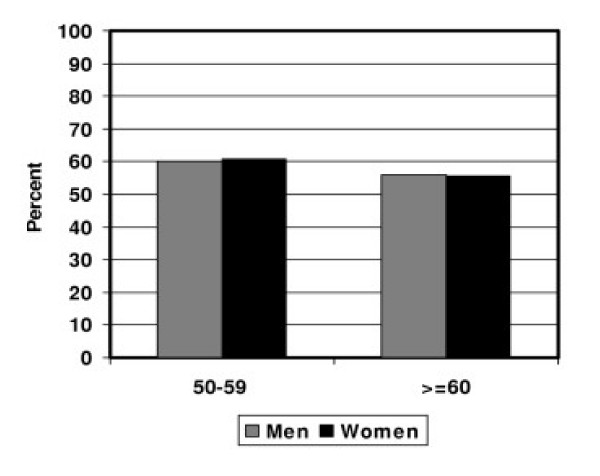
**Colon cancer screening (age 50 & older)**. Graph shows unadjusted weighted percentages of receipt of preventive services by age groups.

**Figure 4:**
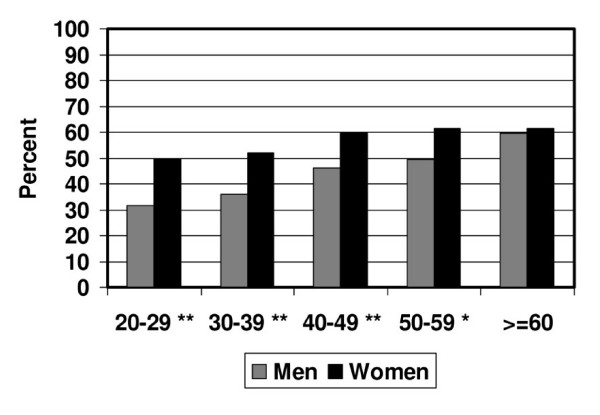
**Counseled to stop smoking in past 12 months**. Graph shows unadjusted weighted percentages of receipt of preventive services by age groups. ** p < .01, * p < .05.

**Figure 5:**
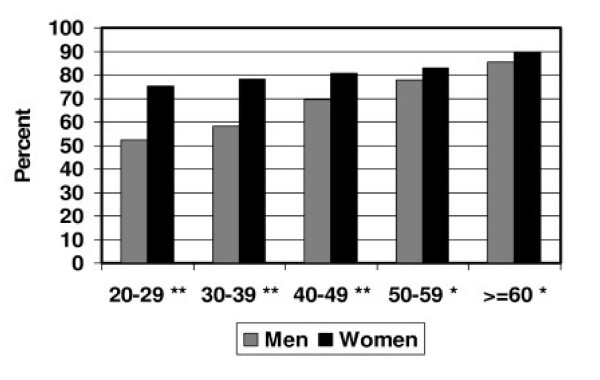
**Checkup within two years**. Graph shows unadjusted weighted percentages of receipt of preventive services by age groups. ** p < .01, * p < .05.

Figures 1, 2, 3, 4, 5 are graphs of unadjusted weighted percentages of men and women stratified by age group who received the selected preventive services. Unadjusted percentages are shown because given the large nationally representative sample used for this study, these measures provide the closest estimates of actual receipt of services. There was no significant difference in receipt of colorectal cancer screening when stratified by age group. All other differences between men and women in receipt of the preventive services within each age stratum were statistically significant with the exception of cholesterol checks in those 60 years and older and smoking cessation counseling in those 60 years and older. For blood pressure checks, the difference between men and women was most pronounced for 20–29 year olds (65% vs. 85%, respectively), whereas this discrepancy narrowed for older ages (87% vs. 91%, for age 60+). Similarly, the difference in receipt of checkups was most pronounced for the youngest group and narrowed for older ages (52% vs. 75% for 20–29 year olds and 86% vs. 89% by age 60+). The graphs for receipt of cholesterol screening and smoking cessation counseling appear to follow a similar pattern.

Table 3 shows adjusted odds ratios for receipt of the selected preventive services by men compared to women stratified by age groups. Although the graphs in the figures suggest interaction between sex and age for all services with the exception of colorectal cancer screening, in adjusted models, there was statistically significant interaction only for blood pressure checks and checkups. For blood pressure checks, the difference between men and women was most pronounced for 20–29 year olds (OR = .38), whereas this discrepancy narrowed for older ages (OR = .68 for age 60+). There was a similar pattern for checkup within two years (OR = .42 for 20–29 and 30–39 year olds vs. OR = .72 for age 60+).

Number of ambulatory care visits in the previous year was added to the original models and interaction between sex and age was again tested. The interaction between sex and age for blood pressure checks was mitigated (p = .206) but overall, men still had lower odds of receiving the service (OR = .58; 95% CI .51–.66). There was no effect on the sex-age interaction for checkups when number of visits was added to the model in this manner.

## Discussion

As shown in previous studies, [6,17] this study confirms that, compared to women, men receive fewer blood pressure measurements and cholesterol measurements. By excluding men and women already diagnosed with hypertension in our analyses of blood pressure measurements and excluding those with hypercholesterolemia in our analyses of cholesterol measurement, we specifically have focused on screening for these health problems. In addition to these findings, we also found that compared to women, a lower percentage of men receive smoking cessation counseling and health checkups. The rates of smoking cessation counseling are notably low in both sexes. Also, as shown elsewhere, colon cancer screening rates are low in both sexes with men receiving colorectal cancer screening at a similar, or slightly higher percentage than women [27,28]. This slight difference may be due to more fear and embarrassment by women about colon endoscopy [29].

The presence of interaction in the adjusted models between sex and age group for blood pressure checks and health checkups indicates that men in the younger age groups are even less likely to receive these services than expected solely by virtue of sex, even after accounting for potential confounders. One might expect the same effect for cholesterol screening, but in combination with adjustment for covariates, the interval of five years may mitigate the effect of interaction by age group. The generally low rates of reported receipt of smoking cessation counseling by both men and women of all ages may have contributed to the lack of interaction on this outcome in the adjusted models. The rates of colorectal cancer screening may not be as affected by age because such screening does not begin until age 50. Men and women 50 years and over may have more similar patterns of health service use than younger men and women. Future research could explore this hypothesis.

It appears that some of the discrepancy in receipt of preventive services between men and women younger than 50 results from the difference in the number of visits to healthcare providers. Previous work supports this finding. For example, one study demonstrated that the rate of visits for annual examinations and preventive services by women was 100% higher than for men [30]. Even after excluding pregnancy-related visits, women were 33% more likely than men to visit a physician, a difference that decreased with age [30]. However, visits should not be thought of as a confounder because, in the current predominant model of preventive healthcare, a visit is a necessary step along the causal pathway to receiving preventive services. In order to increase men's adherence to preventive care recommendations, future research should explicate the motivating factors behind men's decisions to seek outpatient care. Analytically, our findings underscore the importance of using a carefully considered conceptual model to guide analyses of care-seeking behavior. Future research in this area should also consider the use of structural equation modeling techniques to gain a more complete understanding of the complex underlying relationships between variables.

### Limitations

While MEPS is a large, nationally representative survey, the data is cross-sectional and limits our ability to establish causality. More importantly, self-report may not correspond perfectly with receipt of preventive services. Recall bias would be a limitation if recall for the included services differed significantly between men and women or between the young and the old. Finally, operationalizing the number of ambulatory care visits as a dichotomy between one or more and none in the previous twelve months in this analysis assumes care-seeking patterns are reasonably stable from year to year. Therefore, one year would be a close approximation to two years worth of data. However, this method may underestimate the effect of visits on receipt of preventive services and the interaction effect on checkups might indeed be mitigated if two-years of visits were included. Still, this would not change the overall implications of this study.

## Conclusion

With the exception of colorectal cancer screening (for which both men and women have low rates), fewer men receive the preventive services considered in this analysis than women. This disparity is further affected by age group for certain services, such that younger men are particularly vulnerable. This effect may be partly explained by the difference between younger men and women in the number of ambulatory care visits.

Two potential strategies to improve preventive healthcare for young to middle-aged men emerge from this study. One strategy would be to increase visits by these men for ambulatory care. Presumably, such visits would be health maintenance visits. However, this strategy is not ideal because even if men could be persuaded to increase their visits, such a strategy would not only be costly and resource-consuming, but because of disparities in access to care, would also not likely reach those men most likely to benefit. A second strategy would be to develop alternative methods of health promotion and preventive services delivery to younger and middle-aged men. Such strategies might include worksite programs or community programs situated in settings where men live and gather, with appropriate mechanisms to insure contact with health professionals when health problems (e.g., hypertension) are identified.

## Competing interests

The author(s) declare that they have no competing interests.

## Authors' contributions

AJV conceived of the study, performed the analyses, and drafted the initial manuscript and revisions. JMT assisted in revision of conceptual components of the study, provided expertise in MEPS, assistance with MEPS files, assistance with coding, and provided important revisions to the manuscript. JMG provided biostatistical expertise and important contributions to the manuscript, tables, and figures.

## Pre-publication history

The pre-publication history for this paper can be accessed here:



## Figures and Tables

**Table 1:** Characteristics of the sample (n = 14,979*)

Characteristic	Weighted percent^a^	
	
	Men (n = 6916*)	Women (n = 8063*)
Age (years)		
20 – 29	19.4	17.7
30 – 39	21.8	20.9
40 – 49	21.6	21.1
50 – 59	16.5	16.0
60 and over	20.8	24.2
Race/Ethnicity		
Black	10.6	12.4
Hispanic	10.9	10.3
White/other	78.5	77.4
Education		
Less than high school	18.5	18.4
High school graduate	33.1	33.7
Some college	20.7	24.3
College graduate	27.7	23.7
Income		
< $25-000 per year	46.2	60.9
$25-000 – 50-000 per year	33.1	26.6
> $50-000 per year	20.7	12.6
Marital status		
Married	60.8	54.8
Widowed	2.8	11.5
Divorced	10.6	12.5
Separated	1.5	1.9
Never married	24.3	19.3
Insurance		
None	14.0	10.3
Public	11.0	15.8
Private	75.1	73.9
% with usual source of care	73.6	84.0
Perceived health status		
Excellent	19.1	15.4
Very good	37.0	36.3
Good	30.7	31.7
Fair	10.5	13.3
Poor	2.7	3.3
Region of country		
Northeast	19.2	19.4
Midwest	23.6	22.4
South	34.7	36.0
West	22.6	22.2

*Unweighted

^a ^Percentages may not add to exactly 100% due to rounding

**Table 2:** Gender and receipt of basic preventive services in the United States, women as reference group

Preventive service	Odds ratio	95% CI
Blood pressure measured within 2 years (not known hypertensive)		
Unadjusted	.41	.37–.45
Adjusted^a^	.44	.40–.50
Cholesterol checked within 5 years (not known hyperlipidemic)		
Unadjusted	.67	.62–.74
Adjusted^a^	.72	.65–.79
Colorectal cancer screen (if age >= 50)		
Unadjusted	1.22	1.03–1.44
Adjusted^a^	1.16	.98–1.37
Counseled to stop smoking (if smoker)		
Unadjusted	.60	.51–.70
Adjusted^a^	.66	.55–.78
Last routine checkup within 2 years		
Unadjusted	.49	.45–.52
Adjusted^a^	.53	.49–.57

^a^Based on logistic regression models, adjusted for age category, education, race/ethnicity, insurance, usual source of care, income, marital status, perceived health status, and region of country

**Table 3:** Adjusted odds ratios (95% confidence intervals)^a ^of preventive services received by men compared to women

Age (yrs)	Blood pressure within two years	Cholesterol measured within five years	Colorectal cancer screening	Counseled to stop smoking in last year	Checkup in last two years
20–29	.38 (.29–.50)	.70 (.57–.86)	N/A	.62 (.39–1.01)	.42 (.35–.50)
30–39	.40 (.33–.50)	.68 (.57–.80)	N/A	.61 (.44–.86)	.42 (.34–.50)
40–49	.42 (.33–.54)	.79 (.65–.96)	N/A	.65 (.47–.90)	.57 (.49–.67)
50–59	.55 (.41–.75)	.68 (.53–.88)	1.17 (.94–1.46)	.61 (.40–.91)	.75 (.60–.95)
60 & over	.68 (.49–.93)	.75 (.58–.98)	1.14 (.85–1.52)	.89 (.58–1.35)	.72 (.57–.91)
Test for interaction*	p = .016	p = .748	p = .860	p = .614	p < .001

^a^Estimated from logistic regression models including interaction terms between sex and age, and adjusted for age category, education, race/ethnicity, insurance, usual source of care, income, marital status, perceived health status, and region of country.

N/A = not applicable

*P-value based on a 4 degree-of-freedom test of the sex by age interaction effects in each logistic regression model; significant interaction means odds ratios differ across age strata

## References

[B1] (2000). United States Department of Health and Human Services. Healthy People 2010.

[B2] Bindman AB, Grumbach K, Osmond D, Vranizan K, Stewart AL (1996). Primary care and receipt of preventive services. J Gen Intern Med.

[B3] Brown DW, Giles WH, Greenlund KJ, Croft JB (2001). Disparities in cholesterol screening: falling short of a national health objective. Prev Med.

[B4] Carrasquillo O, Lantigua RA, Shea S (2001). Preventive services among Medicare beneficiaries with supplemental coverage versus HMO enrollees, medicaid recipients, and elders with no additional coverage. Med Care.

[B5] Casey MM, Thiede Call K, Klingner JM (2001). Are rural residents less likely to obtain recommended preventive healthcare services?. Am J Prev Med.

[B6] Corbie-Smith G, Flagg EW, Doyle JP, O'Brien MA (2002). Influence of usual source of care on differences by race/ethnicity in receipt of preventive services. J Gen Intern Med.

[B7] DeLaet DE, Shea S, Carrasquillo O (2002). Receipt of preventive services among privately insured minorities in managed care versus fee-for-service insurance plans. J Gen Intern Med.

[B8] DeVoe JE, Fryer GE, Phillips R, Green L (2003). Receipt of preventive care among adults: insurance status and usual source of care. Am J Public Health.

[B9] Doty HE, Weech-Maldonado R (2003). Racial/ethnic disparities in adult preventive dental care use. J Health Care Poor Underserved.

[B10] Faulkner LA, Schauffler HH (1997). The effect of health insurance coverage on the appropriate use of recommended clinical preventive services. Am J Prev Med.

[B11] Haas JS, Phillips KA, Sonneborn D, McCulloch CE, Liang SY (2002). Effect of managed care insurance on the use of preventive care for specific ethnic groups in the United States. Med Care.

[B12] Hueston WJ, Hubbard ET (2000). Preventive services for rural and urban African American adults. Arch Fam Med.

[B13] McIsaac WJ, Fuller-Thomson E, Talbot Y (2001). Does having regular care by a family physician improve preventive care?. Can Fam Physician.

[B14] Sambamoorthi U, McAlpine DD (2003). Racial, ethnic, socioeconomic, and access disparities in the use of preventive services among women. Prev Med.

[B15] Shi R, Berkel H, Sartor O (2000). Comparison of utilization of preventive health care services between two racial populations. Ann Epidemiol.

[B16] Solberg LI, Brekke ML, Kottke TE (1997). Are physicians less likely to recommend preventive services to low-SES patients?. Prev Med.

[B17] Stewart SH, Silverstein MD (2002). Racial and ethnic disparity in blood pressure and cholesterol measurement. J Gen Intern Med.

[B18] Williams RL, Flocke SA, Stange KC (2001). Race and preventive services delivery among black patients and white patients seen in primary care. Med Care.

[B19] Xu KT (2002). Usual source of care in preventive service use: a regular doctor versus a regular site. Health Serv Res.

[B20] Zhang P, Tao G, Irwin KL (2000). Utilization of preventive medical services in the United States: a comparison between rural and urban populations. J Rural Health.

[B21] Finkelstein MM (2002). Preventive screening. What factors influence testing?. Can Fam Physician.

[B22] Medical Expenditure Panel Survey website. http://www.meps.ahrq.gov.

[B23] Andersen RM (1995). Revisiting the behavioral model and access to medical care: does it matter?. J Health Soc Behav.

[B24] USPSTF (2002). Screening for colorectal cancer: recommendation and rationale. Ann Intern Med.

[B25] Kleinbaum DG (1994). Logistic Regression. Statistics in the Health Sciences.

[B26] Hosmer DW, Lemeshow SL, Barnett V, Bradley RA, Hunter JS, Kadane JB, Kendall DG, Smith AFM, Stigler SM and Watson GS (1989). Applied Logistic Regression. Wiley Series in Probability and Mathematical Statistics.

[B27] Walsh JM, Posner SF, Perez-Stable EJ (2002). Colon cancer screening in the ambulatory setting. Prev Med.

[B28] MMWR (2003). Colorectal Cancer Test Use Among Persons Aged >50 Years --- United States, 2001. Morbidity and Mortality Weekly Report.

[B29] Farraye FA, Wong M, Hurwitz S, Puleo E, Emmons K, Wallace MB, Fletcher RH (2004). Barriers to endoscopic colorectal cancer screening: are women different from men?. Am J Gastroenterol.

[B30] Centers for Disease Control. Utilization of ambulatory medical care by women: United States, 1997-98. Series Report 13, No 149.

